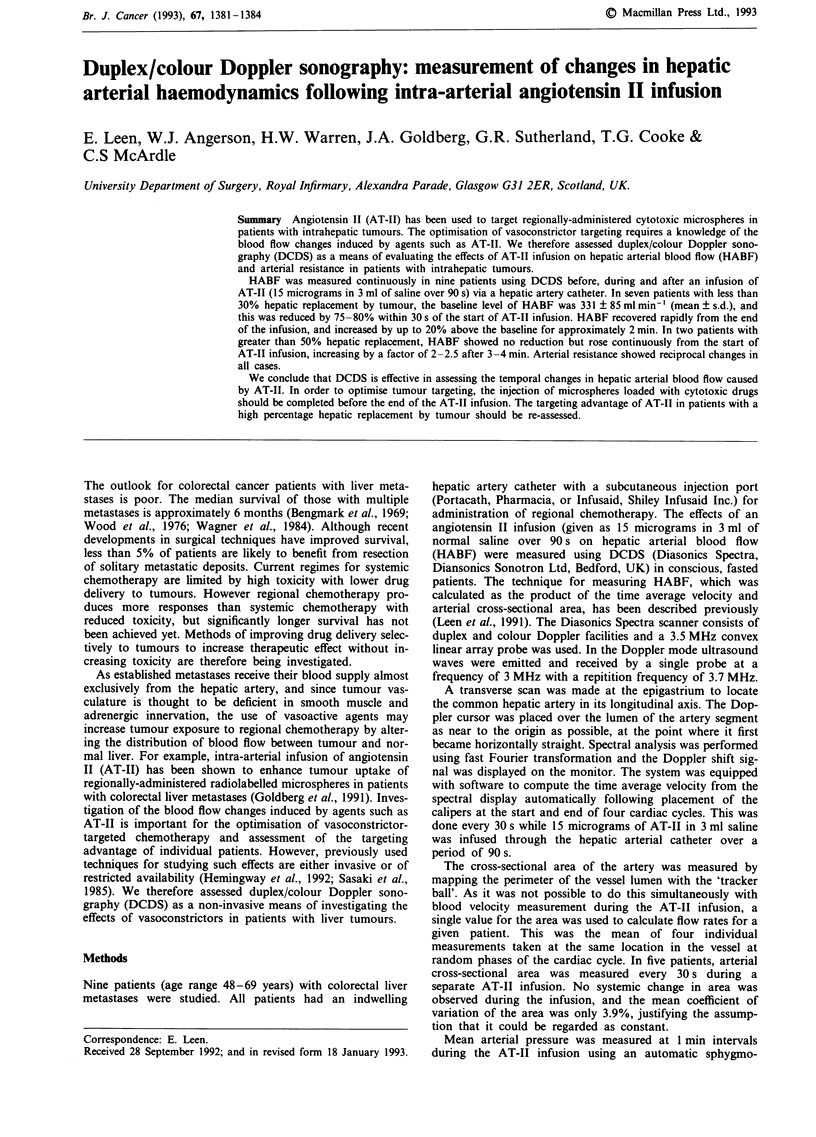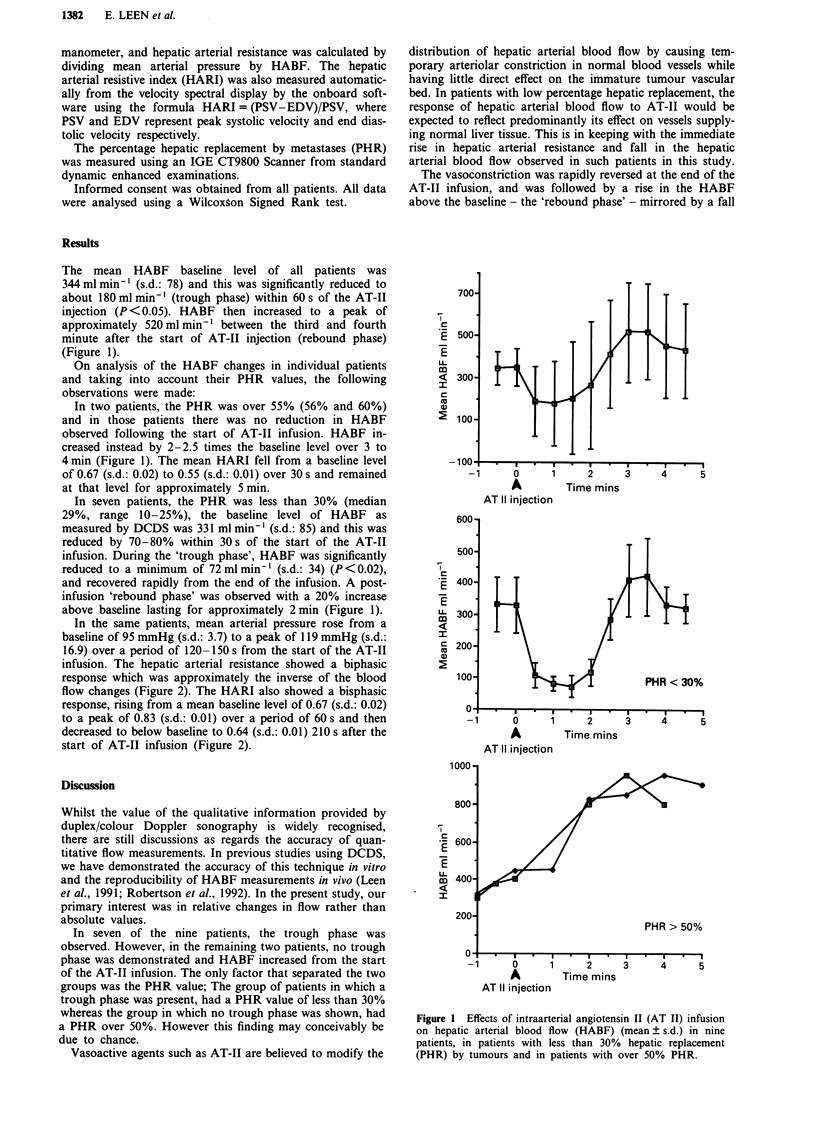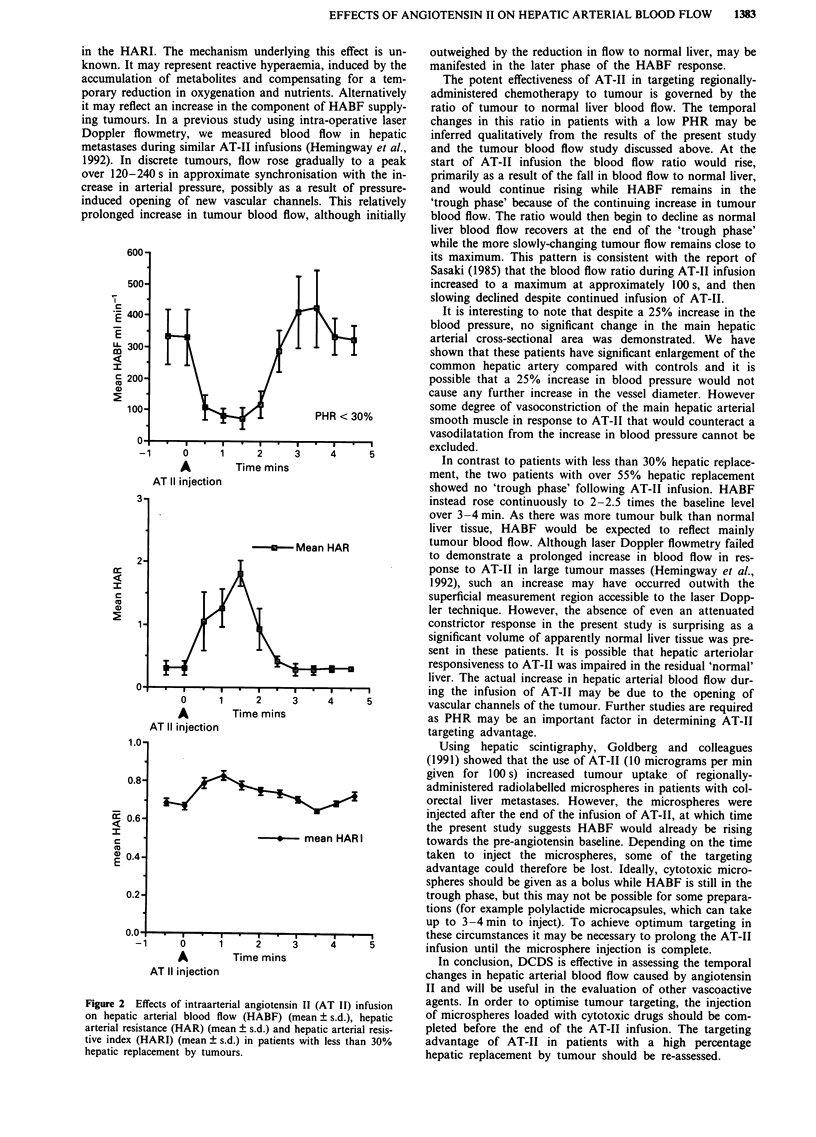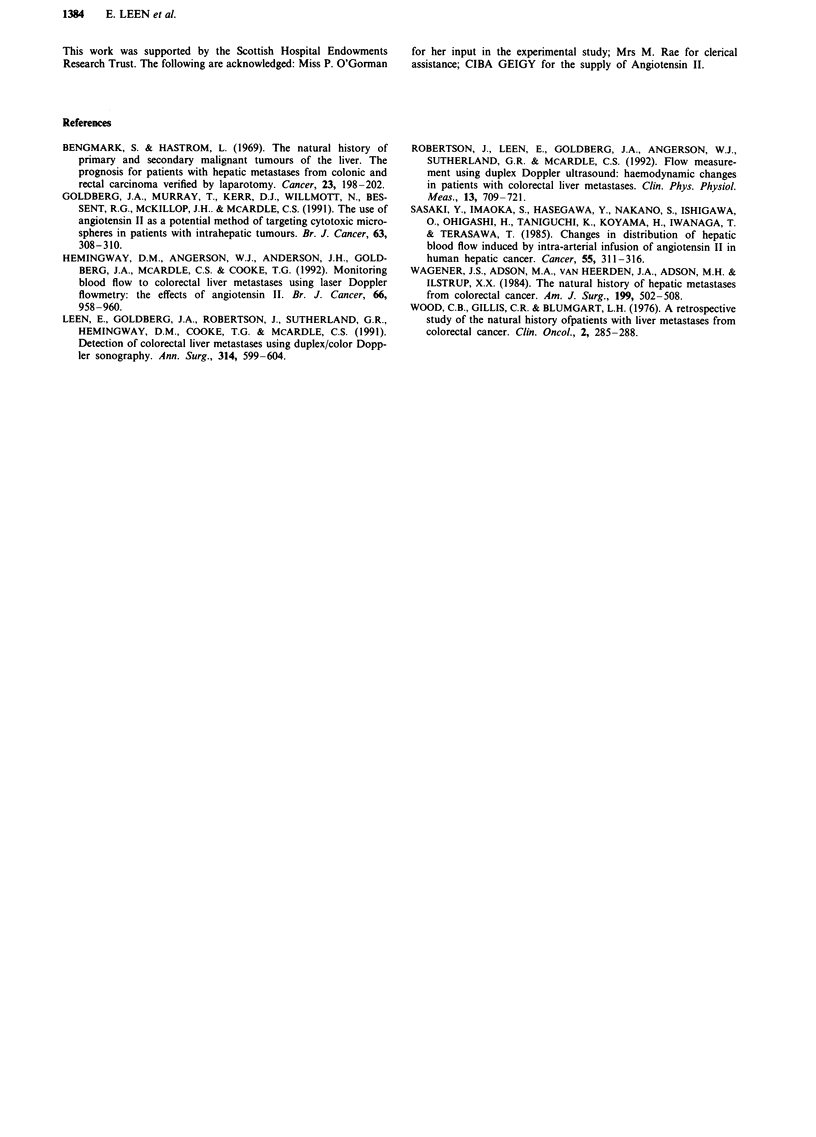# Duplex/colour Doppler sonography: measurement of changes in hepatic arterial haemodynamics following intra-arterial angiotensin II infusion.

**DOI:** 10.1038/bjc.1993.255

**Published:** 1993-06

**Authors:** E. Leen, W. J. Angerson, H. W. Warren, J. A. Goldberg, G. R. Sutherland, T. G. Cooke, C. S. McArdle

**Affiliations:** University Department of Surgery, Royal Infirmary, Glasgow, Scotland, UK.

## Abstract

Angiotensin II (AT-II) has been used to target regionally-administered cytotoxic microspheres in patients with intrahepatic tumours. The optimisation of vasoconstrictor targeting requires a knowledge of the blood flow changes induced by agents such as AT-II. We therefore assessed duplex/colour Doppler sonography (DCDS) as a means of evaluating the effects of AT-II infusion on hepatic arterial blood flow (HABF) and arterial resistance in patients with intrahepatic tumours. HABF was measured continuously in nine patients using DCDS before, during and after an infusion of AT-II (15 micrograms in 3 ml of saline over 90 s) via a hepatic artery catheter. In seven patients with less than 30% hepatic replacement by tumour, the baseline level of HABF was 331 +/- 85 ml min-1 (mean +/- s.d.), and this was reduced by 75-80% within 30 s of the start of AT-II infusion. HABF recovered rapidly from the end of the infusion, and increased by up to 20% above the baseline for approximately 2 min. In two patients with greater than 50% hepatic replacement, HABF showed no reduction but rose continuously from the start of AT-II infusion, increasing by a factor of 2-2.5 after 3-4 min. Arterial resistance showed reciprocal changes in all cases. We conclude that DCDS is effective in assessing the temporal changes in hepatic arterial blood flow caused by AT-II. In order to optimise tumour targeting, the injection of microspheres loaded with cytotoxic drugs should be completed before the end of the AT-II infusion. The targeting advantage of AT-II in patients with a high percentage hepatic replacement by tumour should be re-assessed.


Br. J. Cancer (1993), 67, 1381–1384 © Macmillan Press Ltd., 1993

### Duplex/colour Doppler sonography: measurement of changes in hepatic arterial haemodynamics following intra-arterial angiotensin II infusion

E. Leen, W.J. Angerson, H.W. Warren, J.A. Goldberg, G.R. Sutherland, T.G. Cooke & C.S McArdle

*University Department of Surgery, Royal Infirmary, Alexandra Parade, Glasgow G31 2ER, Scotland, UK.*

**Summary** Angiotensin II (AT-II) has been used to target regionally-administered cytotoxic microspheres in patients with intrahepatic tumours. The optimisation of vasoconstrictor targeting requires a knowledge of the blood flow changes induced by agents such as AT-II. We therefore assessed duplex/colour Doppler sonography (DCDS) as a means of evaluating the effects of AT-II infusion on hepatic arterial blood flow (HABF) and arterial resistance in patients with intrahepatic tumours.

HABF was measured continuously in nine patients using DCDS before, during and after an infusion of AT-II (15 micrograms in 3 ml of saline over 90 s) via a hepatic artery catheter. In seven patients with less than 30% hepatic replacement by tumour, the baseline level of HABF was 331 ± 85 ml min$^{-1}$ (mean ± s.d.), and this was reduced by 75–80% within 30 s of the start of AT-II infusion. HABF recovered rapidly from the end of the infusion, and increased by up to 20% above the baseline for approximately 2 min. In two patients with greater than 50% hepatic replacement, HABF showed no reduction but rose continuously from the start of AT-II infusion, increasing by a factor of 2–2.5 after 3–4 min. Arterial resistance showed reciprocal changes in all cases.

We conclude that DCDS is effective in assessing the temporal changes in hepatic arterial blood flow caused by AT-II. In order to optimise tumour targeting, the injection of microspheres loaded with cytotoxic drugs should be completed before the end of the AT-II infusion. The targeting advantage of AT-II in patients with a high percentage hepatic replacement by tumour should be re-assessed.

The outlook for colorectal cancer patients with liver metastases is poor. The median survival of those with multiple metastases is approximately 6 months (Bengmark *et al.*, 1969; Wood *et al.*, 1976; Wagner *et al.*, 1984). Although recent developments in surgical techniques have improved survival, less than 5% of patients are likely to benefit from resection of solitary metastatic deposits. Current regimes for systemic chemotherapy are limited by high toxicity with lower drug delivery to tumours. However regional chemotherapy produces more responses than systemic chemotherapy with reduced toxicity, but significantly longer survival has not been achieved yet. Methods of improving drug delivery selectively to tumours to increase therapeutic effect without increasing toxicity are therefore being investigated.

As established metastases receive their blood supply almost exclusively from the hepatic artery, and since tumour vasculature is thought to be deficient in smooth muscle and adrenergic innervation, the use of vasoactive agents may increase tumour exposure to regional chemotherapy by altering the distribution of blood flow between tumour and normal liver. For example, intra-arterial infusion of angiotensin II (AT-II) has been shown to enhance tumour uptake of regionally-administered radiolabelled microspheres in patients with colorectal liver metastases (Goldberg *et al.*, 1991). Investigation of the blood flow changes induced by agents such as AT-II is important for the optimisation of vasoconstrictor-targeted chemotherapy and assessment of the targeting advantage of individual patients. However, previously used techniques for studying such effects are either invasive or of restricted availability (Hemingway *et al.*, 1992; Sasaki *et al.*, 1985). We therefore assessed duplex/colour Doppler sonography (DCDS) as a non-invasive means of investigating the effects of vasoconstrictors in patients with liver tumours.

### Methods

Nine patients (age range 48–69 years) with colorectal liver metastases were studied. All patients had an indwelling hepatic artery catheter with a subcutaneous injection port (Portacath, Pharmacia, or Infusaid, Shiley Infusaid Inc.) for administration of regional chemotherapy. The effects of an angiotensin II infusion (given as 15 micrograms in 3 ml of normal saline over 90 s on hepatic arterial blood flow (HABF) were measured using DCDS (Diasonics Spectra, Diansonics Sonotron Ltd, Bedford, UK) in conscious, fasted patients. The technique for measuring HABF, which was calculated as the product of the time average velocity and arterial cross-sectional area, has been described previously (Leen *et al.*, 1991). The Diasonics Spectra scanner consists of duplex and colour Doppler facilities and a 3.5 MHz convex linear array probe was used. In the Doppler mode ultrasound waves were emitted and received by a single probe at a frequency of 3 MHz with a repitition frequency of 3.7 MHz.

A transverse scan was made at the epigastrium to locate the common hepatic artery in its longitudinal axis. The Doppler cursor was placed over the lumen of the artery segment as near to the origin as possible, at the point where it first became horizontally straight. Spectral analysis was performed using fast Fourier transformation and the Doppler shift signal was displayed on the monitor. The system was equipped with software to compute the time average velocity from the spectral display automatically following placement of the calipers at the start and end of four cardiac cycles. This was done every 30 s while 15 micrograms of AT-II in 3 ml saline was infused through the hepatic arterial catheter over a period of 90 s.

The cross-sectional area of the artery was measured by mapping the perimeter of the vessel lumen with the 'tracker ball'. As it was not possible to do this simultaneously with blood velocity measurement during the AT-II infusion, a single value for the area was used to calculate flow rates for a given patient. This was the mean of four individual measurements taken at the same location in the vessel at random phases of the cardiac cycle. In five patients, arterial cross-sectional area was measured every 30 s during a separate AT-II infusion. No systemic change in area was observed during the infusion, and the mean coefficient of variation of the area was only 3.9%, justifying the assumption that it could be regarded as constant.

Mean arterial pressure was measured at 1 min intervals during the AT-II infusion using an automatic sphygmo-

Correspondence: E. Leen.
Received 28 September 1992; and in revised form 18 January 1993.

manometer, and hepatic arterial resistance was calculated by dividing mean arterial pressure by HABF. The hepatic arterial resistive index (HARI) was also measured automatically from the velocity spectral display by the onboard software using the formula HARI = (PSV − EDV)/PSV, where PSV and EDV represent peak systolic velocity and end diastolic velocity respectively.

The percentage hepatic replacement by metastases (PHR) was measured using an IGE CT9800 Scanner from standard dynamic enhanced examinations.

Informed consent was obtained from all patients. All data were analysed using a Wilcoxŝon Signed Rank test.

### Results

The mean HABF baseline level of all patients was 344 ml min$^{-1}$ (s.d.: 78) and this was significantly reduced to about 180 ml min$^{-1}$ (trough phase) within 60 s of the AT-II injection ($P<0.05$). HABF then increased to a peak of approximately 520 ml min$^{-1}$ between the third and fourth minute after the start of AT-II injection (rebound phase) (Figure 1).

On analysis of the HABF changes in individual patients and taking into account their PHR values, the following observations were made:

In two patients, the PHR was over 55% (56% and 60%) and in those patients there was no reduction in HABF observed following the start of AT-II infusion. HABF increased instead by 2–2.5 times the baseline level over 3 to 4 min (Figure 1). The mean HARI fell from a baseline level of 0.67 (s.d.: 0.02) to 0.55 (s.d.: 0.01) over 30 s and remained at that level for approximately 5 min.

In seven patients, the PHR was less than 30% (median 29%, range 10–25%), the baseline level of HABF as measured by DCDS was 331 ml min$^{-1}$ (s.d.: 85) and this was reduced by 70–80% within 30 s of the start of the AT-II infusion. During the 'trough phase', HABF was significantly reduced to a minimum of 72 ml min$^{-1}$ (s.d.: 34) ($P<0.02$), and recovered rapidly from the end of the infusion. A post-infusion 'rebound phase' was observed with a 20% increase above baseline lasting for approximately 2 min (Figure 1).

In the same patients, mean arterial pressure rose from a baseline of 95 mmHg (s.d.: 3.7) to a peak of 119 mmHg (s.d.: 16.9) over a period of 120–150 s from the start of the AT-II infusion. The hepatic arterial resistance showed a biphasic response which was approximately the inverse of the blood flow changes (Figure 2). The HARI also showed a bisphasic response, rising from a mean baseline level of 0.67 (s.d.: 0.02) to a peak of 0.83 (s.d.: 0.01) over a period of 60 s and then decreased to below baseline to 0.64 (s.d.: 0.01) 210 s after the start of AT-II infusion (Figure 2).

### Discussion

Whilst the value of the qualitative information provided by duplex/colour Doppler sonography is widely recognised, there are still discussions as regards the accuracy of quantitative flow measurements. In previous studies using DCDS, we have demonstrated the accuracy of this technique *in vitro* and the reproducibility of HABF measurements *in vivo* (Leen *et al.*, 1991; Robertson *et al.*, 1992). In the present study, our primary interest was in relative changes in flow rather than absolute values.

In seven of the nine patients, the trough phase was observed. However, in the remaining two patients, no trough phase was demonstrated and HABF increased from the start of the AT-II infusion. The only factor that separated the two groups was the PHR value; The group of patients in which a trough phase was present, had a PHR value of less than 30% whereas the group in which no trough phase was shown, had a PHR over 50%. However this finding may conceivably be due to chance.

Vasoactive agents such as AT-II are believed to modify the distribution of hepatic arterial blood flow by causing temporary arteriolar constriction in normal blood vessels while having little direct effect on the immature tumour vascular bed. In patients with low percentage hepatic replacement, the response of hepatic arterial blood flow to AT-II would be expected to reflect predominantly its effect on vessels supplying normal liver tissue. This is in keeping with the immediate rise in hepatic arterial resistance and fall in the hepatic arterial blood flow observed in such patients in this study.

The vasoconstriction was rapidly reversed at the end of the AT-II infusion, and was followed by a rise in the HABF above the baseline – the 'rebound phase' – mirrored by a fall

**Figure 1** Effects of intraarterial angiotensin II (AT II) infusion on hepatic arterial blood flow (HABF) (mean ± s.d.) in nine patients, in patients with less than 30% hepatic replacement (PHR) by tumours and in patients with over 50% PHR.

in the HARI. The mechanism underlying this effect is unknown. It may represent reactive hyperaemia, induced by the accumulation of metabolites and compensating for a temporary reduction in oxygenation and nutrients. Alternatively it may reflect an increase in the component of HABF supplying tumours. In a previous study using intra-operative laser Doppler flowmetry, we measured blood flow in hepatic metastases during similar AT-II infusions (Hemingway *et al.*, 1992). In discrete tumours, flow rose gradually to a peak over 120–240 s in approximate synchronisation with the increase in arterial pressure, possibly as a result of pressure-induced opening of new vascular channels. This relatively prolonged increase in tumour blood flow, although initially

**Figure 2** Effects of intraarterial angiotensin II (AT II) infusion on hepatic arterial blood flow (HABF) (mean ± s.d.), hepatic arterial resistance (HAR) (mean ± s.d.) and hepatic arterial resistive index (HARI) (mean ± s.d.) in patients with less than 30% hepatic replacement by tumours.

outweighed by the reduction in flow to normal liver, may be manifested in the later phase of the HABF response.

The potent effectiveness of AT-II in targeting regionally-administered chemotherapy to tumour is governed by the ratio of tumour to normal liver blood flow. The temporal changes in this ratio in patients with a low PHR may be inferred qualitatively from the results of the present study and the tumour blood flow study discussed above. At the start of AT-II infusion the blood flow ratio would rise, primarily as a result of the fall in blood flow to normal liver, and would continue rising while HABF remains in the 'trough phase' because of the continuing increase in tumour blood flow. The ratio would then begin to decline as normal liver blood flow recovers at the end of the 'trough phase' while the more slowly-changing tumour flow remains close to its maximum. This pattern is consistent with the report of Sasaki (1985) that the blood flow ratio during AT-II infusion increased to a maximum at approximately 100 s, and then slowing declined despite continued infusion of AT-II.

It is interesting to note that despite a 25% increase in the blood pressure, no significant change in the main hepatic arterial cross-sectional area was demonstrated. We have shown that these patients have significant enlargement of the common hepatic artery compared with controls and it is possible that a 25% increase in blood pressure would not cause any further increase in the vessel diameter. However some degree of vasoconstriction of the main hepatic arterial smooth muscle in response to AT-II that would counteract a vasodilatation from the increase in blood pressure cannot be excluded.

In contrast to patients with less than 30% hepatic replacement, the two patients with over 55% hepatic replacement showed no 'trough phase' following AT-II infusion. HABF instead rose continuously to 2–2.5 times the baseline level over 3–4 min. As there was more tumour bulk than normal liver tissue, HABF would be expected to reflect mainly tumour blood flow. Although laser Doppler flowmetry failed to demonstrate a prolonged increase in blood flow in response to AT-II in large tumour masses (Hemingway *et al.*, 1992), such an increase may have occurred outwith the superficial measurement region accessible to the laser Doppler technique. However, the absence of even an attenuated constrictor response in the present study is surprising as a significant volume of apparently normal liver tissue was present in these patients. It is possible that hepatic arteriolar responsiveness to AT-II was impaired in the residual 'normal' liver. The actual increase in hepatic arterial blood flow during the infusion of AT-II may be due to the opening of vascular channels of the tumour. Further studies are required as PHR may be an important factor in determining AT-II targeting advantage.

Using hepatic scintigraphy, Goldberg and colleagues (1991) showed that the use of AT-II (10 micrograms per min given for 100 s) increased tumour uptake of regionally-administered radiolabelled microspheres in patients with colorectal liver metastases. However, the microspheres were injected after the end of the infusion of AT-II, at which time the present study suggests HABF would already be rising towards the pre-angiotensin baseline. Depending on the time taken to inject the microspheres, some of the targeting advantage could therefore be lost. Ideally, cytotoxic microspheres should be given as a bolus while HABF is still in the trough phase, but this may not be possible for some preparations (for example polylactide microcapsules, which can take up to 3–4 min to inject). To achieve optimum targeting in these circumstances it may be necessary to prolong the AT-II infusion until the microsphere injection is complete.

In conclusion, DCDS is effective in assessing the temporal changes in hepatic arterial blood flow caused by angiotensin II and will be useful in the evaluation of other vascoactive agents. In order to optimise tumour targeting, the injection of microspheres loaded with cytotoxic drugs should be completed before the end of the AT-II infusion. The targeting advantage of AT-II in patients with a high percentage hepatic replacement by tumour should be re-assessed.

This work was supported by the Scottish Hospital Endowments Research Trust. The following are acknowledged: Miss P. O'Gorman for her input in the experimental study; Mrs M. Rae for clerical assistance; CIBA GEIGY for the supply of Angiotensin II.

### References

BENGMARK, S. & HASTROM, L. (1969). The natural history of primary and secondary malignant tumours of the liver. The prognosis for patients with hepatic metastases from colonic and rectal carcinoma verified by laparotomy. *Cancer*, **23**, 198–202.

GOLDBERG, J.A., MURRAY, T., KERR, D.J., WILLMOTT, N., BESSENT, R.G., MCKILLOP, J.H.. & MCARDLE, C.S. (1991). The use of angiotensin II as a potential method of targeting cytotoxic microspheres in patients with intrahepatic tumours. *Br. J. Cancer*, **63**, 308–310.

HEMINGWAY, D.M., ANGERSON, W.J., ANDERSON, J.H., GOLDBERG, J.A., MCARDLE, C.S. & COOKE, T.G. (1992). Monitoring blood flow to colorectal liver metastases using laser Doppler flowmetry: the effects of angiotensin II. *Br. J. Cancer*, **66**, 958–960.

LEEN, E., GOLDBERG, J.A., ROBERTSON, J., SUTHERLAND, G.R., HEMINGWAY, D.M., COOKE, T.G. & MCARDLE, C.S. (1991). Detection of colorectal liver metastases using duplex/color Doppler sonography. *Ann. Surg.*, **314**, 599–604.

ROBERTSON, J., LEEN, E., GOLDBERG, J.A., ANGERSON, W.J., SUTHERLAND, G.R. & MCARDLE, C.S. (1992). Flow measurement using duplex Doppler ultrasound: haemodynamic changes in patients with colorectal liver metastases. *Clin. Phys. Physiol. Meas.*, **13**, 709–721.

SASAKI, Y., IMAOKA, S., HASEGAWA, Y., NAKANO, S., ISHIGAWA, O., OHIGASHI, H., TANIGUCHI, K., KOYAMA, H., IWANAGA, T. & TERASAWA, T. (1985). Changes in distribution of hepatic blood flow induced by intra-arterial infusion of angiotensin II in human hepatic cancer. *Cancer*, **55**, 311–316.

WAGENER, J.S., ADSON, M.A., VAN HEERDEN, J.A., ADSON, M.H. & ILSTRUP, X.X. (1984). The natural history of hepatic metastases from colorectal cancer. *Am. J. Surg.*, **199**, 502–508.

WOOD, C.B., GILLIS, C.R. & BLUMGART, L.H. (1976). A retrospective study of the natural history ofpatients with liver metastases from colorectal cancer. *Clin. Oncol.*, **2**, 285–288.